# Supplementary material for: Ballistic‐Aggregated Carbon Nanofoam in Target‐Side of Pulsed Laser Deposition for Energy Storage Applications
Source: ChemSusChem. 2024 Sep 6;17(24):e202400755. doi: 10.1002/cssc.202400755 (PMC11660740; doi:10.1002/cssc.202400755)
Supplement: Supplementary file 1 — Supporting Information [file CSSC-17-e202400755-s001.pdf]

ChemSusChem

Supporting Information

Ballistic-Aggregated Carbon Nanofoam in Target-Side of Pulsed Laser Deposition for Energy Storage Applications

Subrata Ghosh,* Massimiliano Righi, Andrea Macrelli, Giorgio Divitini, Davide Orecchia, Alessandro Maffini, Francesco Goto, Gianlorenzo Bussetti, David Dellasega, Valeria Russo, Andrea Li Bassi, and Carlo S. Casari*

SUPPORTING INFORMATION

Ballistic-aggregated Amorphous Carbon Nanofoam in Target side of Pulsed Laser Deposition for Energy Storage Applications

Subrata Ghosh[1*], Massimiliano Righi[1], Andrea Macrelli[1], Giorgio Divitini,[2] Davide Orecchia[1], Alessandro Maffini[1], Francesco Goto[3], Gianlorenzo Bussetti[3], David Dellasega[1], Valeria Russo[1], Andrea Li Bassi[1], Carlo S. Casari[1*]

[1] *Micro and Nanostructured Materials Laboratory — NanoLab, Department of Energy, Politecnico di Milano, via Ponzio 34/3, Milano, 20133, Italy*

[2] *Electron Spectroscopy and Nanoscopy, Istituto Italiano di Tecnologia, via Morego 30, Genova, 16163, Italy*

[3] *Solid Liquid Interface Nano-Microscopy and Spectroscopy (SoLINano-Σ) lab, Department of Physics, Politecnico di Milano, Piazza Leonardo da Vinci 32, 20133 Milano, Italy*

Corresponding author email: subrata.ghosh@polimi.it (S.G.) and carlo.casari@polimi.it (C.S.C.)

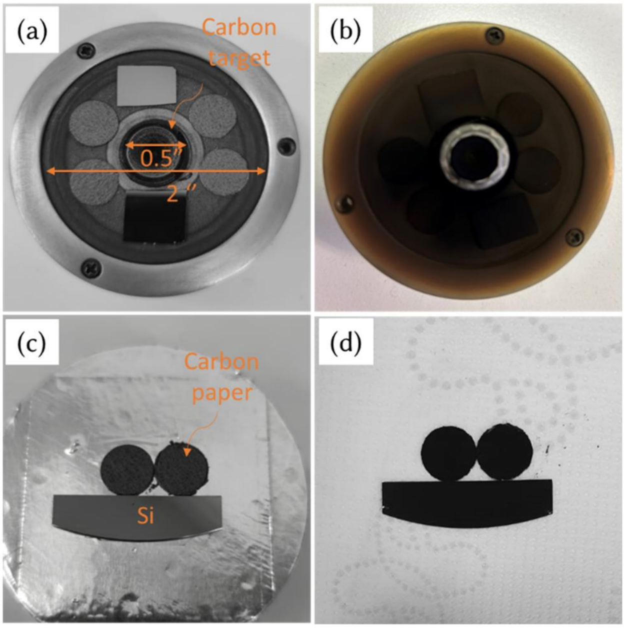

Figure S1: Photograph of target holder (a) before and (b) after carbon nanofoam deposition. Photograph of (c) substrate holder with the substrate before deposition and (d) substrate after deposition.

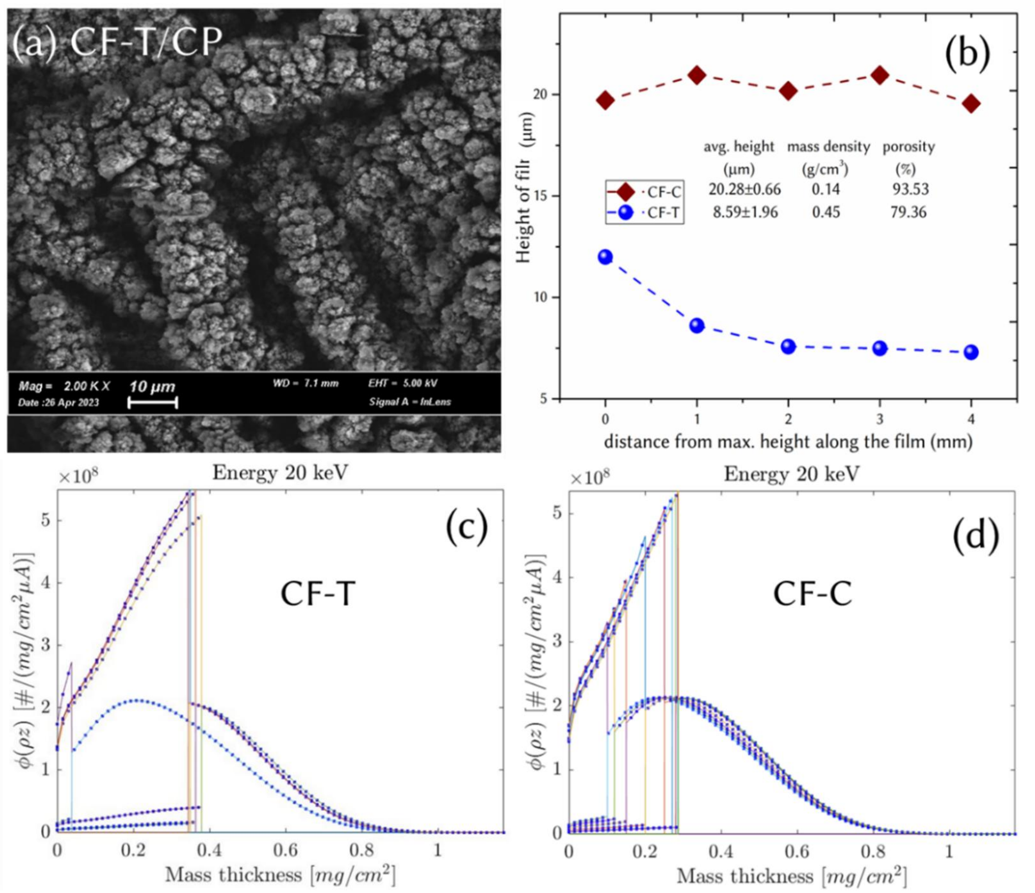

Figure S2: (a) scanning electron micrograph of CF-T grown on carbon paper, CP. (b) plot of height of the CF-T and CF-C film. Quantified average height, mass density and porosity of the films are provided in the inset of plot. (b) Simulated data of mass-density calculation of carbon nanofoam (c) CF-T and (d) CF-C using EDDIE software. The y-axis of plot (c-d) represent X-ray generation distribution function (ϕ(ρz)) in samples and each colored line corresponds to the iteration.

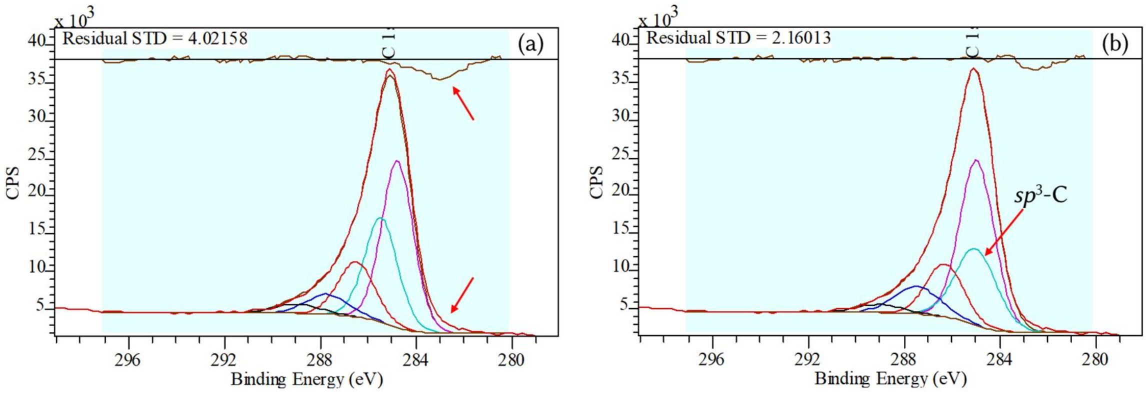

Figure S3: Casa XPS fitted C1*s* spectra without considering the peak *sp*-C at binding energy at around 283.8 eV. (a) Deconvoluted C1*s* spectra without *sp*-C peak shows higher residual STD of 4.02158. (b) fitted C1*s* spectra with deconvoluted peaks and without *sp*-C. In this fit, one peak (*sp*³-C peak – sky blue color) lies under another peak (*sp*²-C peak - pink colour). This fitting is not scientifically reliable. In the figure CPS stand for count per second and STD stand for standard deviation.

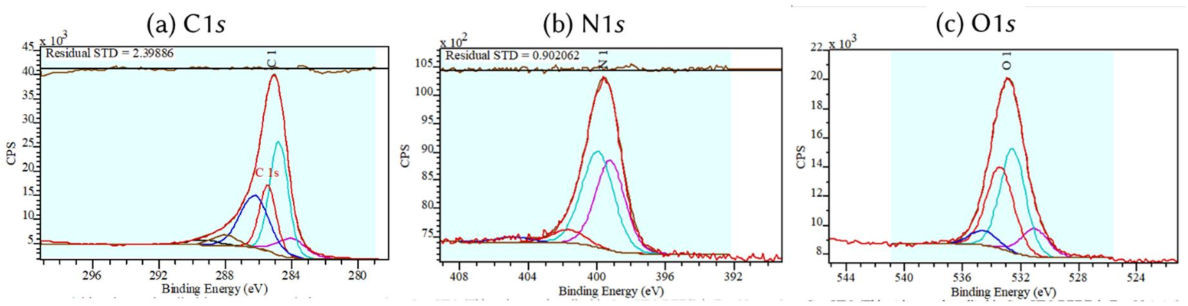

Figure S4. Casa XPS fitted High resolution (a) C1s, (b) N1s and (c) O1s spectra with deconvoluted peaks of CF-C.

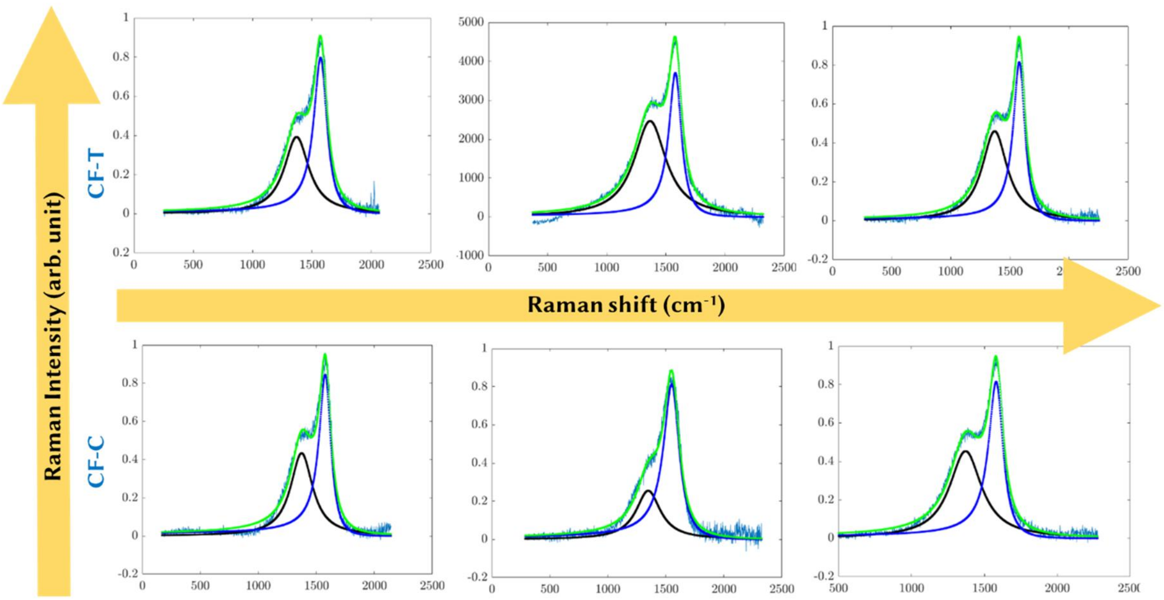

Figure S5: Fitted 1st-order Raman spectra of (top panel) CF-T and (bottom panel) CF-C taken at different position/samples using the MATLAB program. D-peak is Lorentzian shape and G-peak is fitted with BWF-lineshape

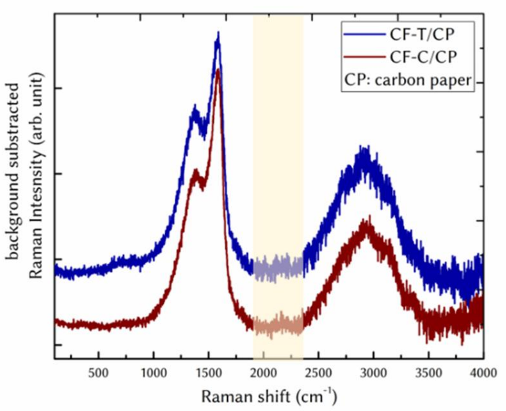

Figure S6: Raman spectra of CF-T and CF-C on carbon paper. A very weak *sp*-band is also observed for the sample on carbon paper, which is marked by highlighting region along with the other prominent peaks.

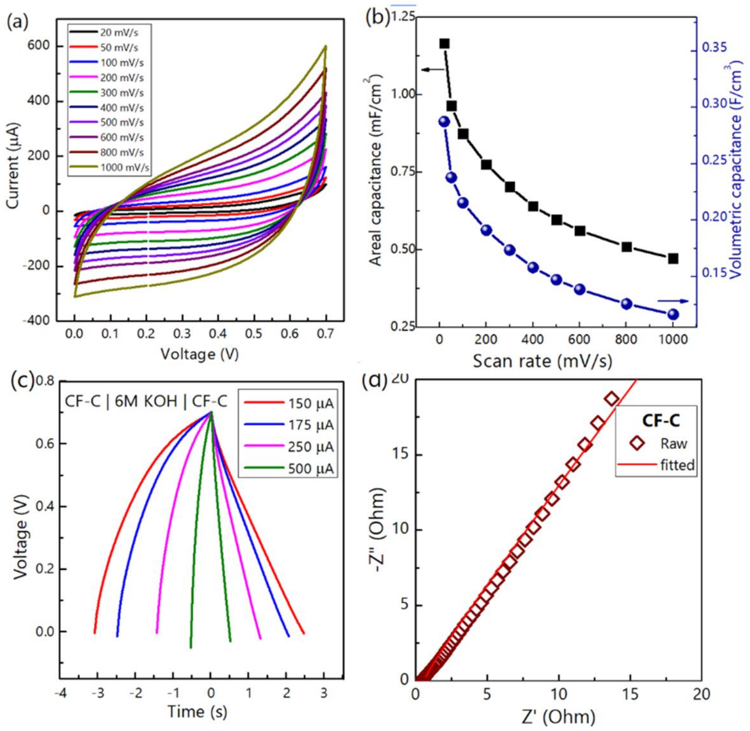

Figure S7: Electrochemical performance of CF-C symmetric device. (a) cyclic voltammogram at different scan rates, (b) areal and volumetric capacitance with respect to scan rate. (c) charge-discharge profile at different current. (d) Nyquist plot and fitted data with equivalent electric circuit.

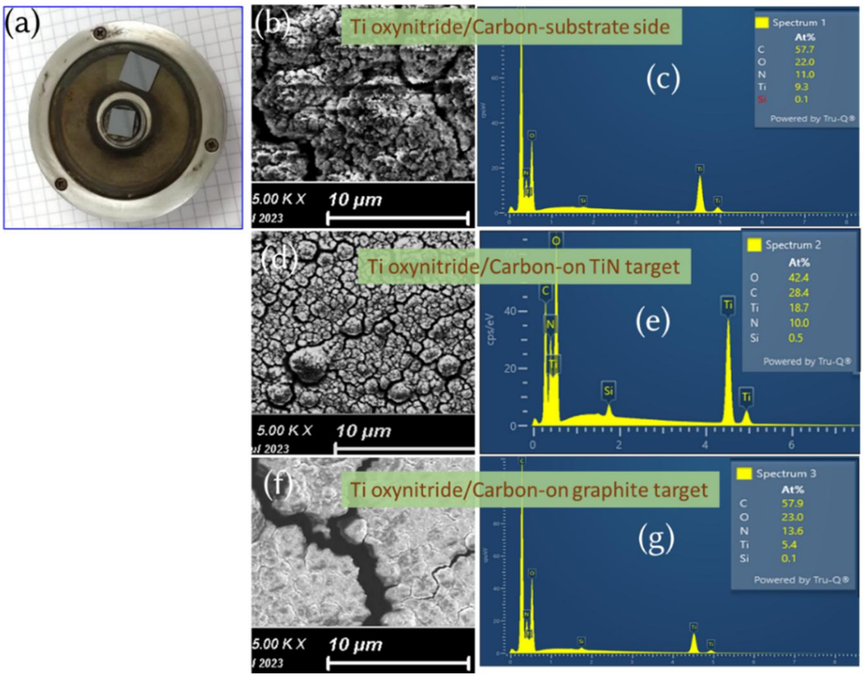

Figure S8: (a) Optical micrograph of the target side, where a Si substrate is placed on the graphite target and the TiN target. Scanning electron micrograph and corresponding EDX spectra of oxynitride/carbon composite prepared on the (b-c) substrate-side, (d-e) on TiN in target side and (f-g) on graphite in target side.
